# Lignin, an active component in the corn silk water extract, inhibits glycation

**DOI:** 10.1038/s41598-022-21780-6

**Published:** 2022-10-22

**Authors:** Aiko Sano, Yutaka Inoue, Ryuichiro Suzuki

**Affiliations:** Faculty of Pharmacy and Pharmaceutical Sciences, Josai University, 1-1 Keyakidai, Sakado, Saitama 350-0295 Japan

**Keywords:** Secondary metabolism, Analytical chemistry, Natural products

## Abstract

The formation of advanced glycation end products is associated with aging and diabetic complications such as neuropathy, retinopathy, and nephropathy. Thus, the suppression of AGEs formation could prevent and/or treat their related disorders. Corn silk is used as a traditional medicine for the prevention of diabetic complications and treatment of edema in Japan and China. Previous studies revealed the anti-glycation activity of flavonoids in the methanolic extract of corn silk. The anti-glycation activity of the corn silk water extract was higher than that of the methanolic extract; however, the active components of the water extract remained unidentified. The purpose of this study is to make clear the components showing anti-glycation activity in the corn silk water extract and elucidated their structural characteristics. The evaluation of anti-glycation activity was carried out by enzyme-linked immunosorbent assay to detect glycated bovine serum albumin. Remarkable anti-glycation activity was observed in the > 3 kDa fraction. Reversed-phase HPLC analysis of this fraction showed broad peaks characteristic of high-molecular-weight polyphenols. Decomposition reactions did not provide evidence of condensed or acid-hydrolyzable tannins. Therefore, polyphenols contained in the corn silk water extract were considered to be lignin-carbohydrate complex. The ^1^H- and ^13^C-nuclear magnetic resonance (NMR) and Fourier transform infrared (FT-IR) spectroscopy spectra of the > 3 kDa fraction were in agreement with the values reported for lignin. Consequently, we concluded that lignin-carbohydrate complex is one of the active components against glycation in the corn silk water extract.

## Introduction

Reducing sugars such as glucose can undergo several non-enzymatically reactions with the amino groups in proteins, lipids, and nucleic acids, forming Schiff bases and Amadori products to produce advanced glycation end products (AGEs). The process of AGEs formation is known as glycation. Some AGEs, including *N*^ε^-(carboxymethyl) lysine (CML) and pentosidine, have been detected in the lesions of patients with diabetes^1^. AGEs formation affects the function and half-life of proteins and induces protein denaturation and irreversible damage. Moreover, AGEs formations associated with the development of diabetic complications, such as neurological, cardiovascular, and renal dysfunctions^2,3^. The rate of formation of AGEs increases under diabetic conditions and glycation is one of the major contributing pathways responsible for the formation of AGEs. Glycation has been found to be a significant causative factor for several health-related issues such as antherosclerosis, chronic renal failure, vascular disease, neurodegerative diseases and aging. The formation of AGEs is accompanied by the generation of various free radicals and reactive oxygen species (ROS) and induces oxidative stress. ROS have been implicated in the pathophysiology of several serious diseases such as aging, inflammation, cancer, and diabetes complications^4^. Therefore, inhibitors of AGEs formation have recently received increasing attention as potential therapeutic agents.

Currently, many glycation inhibitors derived from synthetic and natural compounds have been reported^5^. Aminoguanidine, a hydrazine-mimetic compound, is a well-known synthetic glycation inhibitor, whereas the flavonoids such as rutin and luteolin are natural glycation inhibitors. Some glycation inhibitors might act by interfering with the attachment of sugars with proteins, by inhibiting the late stage of glycation or by preventing Amadori product formation. Furthermore, they might scavenge free radicals and break cross-linkage of proteins caused by glycation^6^. Fruits, vegetables, and beverages are major sources of naturally occurring flavonoids and are relevant for the discovery of new glycation inhibitors^7^. Natural compounds exist abundantly in nature, and their structures are complex and diverse. These natural compounds have many physiological and pharmacological activities. As the results, natural products could be suitable for therapeutic agents against lifestyle-related diseases such as diabetic complications induced by various factors. Natural compounds are also inexpensive, readily available, and easy to consume. Furthermore, they can be used not only for treatment but also for prevention. By the way, corn silk contains vitamins, proteins, sodium salts, volatile oils, steroids, saponins, tannins, and flavonoids. Corn silk refers to the stigmas of corn (*Zea mays* L., Gramineae) and has been used in folk medicine as a diuretic and for the prevention of diabetic complications in Japan and China^8–10^.

**Figure 1:**
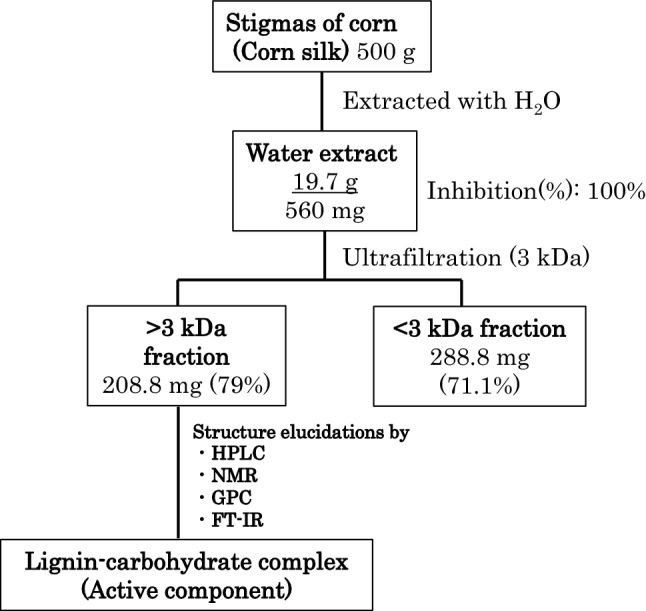
The overview of experimental procedure of this research.

In our previous study, we observed the anti-glycation activity of corn silk. Methanolic extract of corn silk inhibited reaction of glucose with bovine serum albumin (BSA) to generate glycated albumin. Serum albumin such as BSA acts as binding and transport protein for endogenous and exogenous ligand. Glycation of serum albumin leads to loss of its function. It is known that glucose selectively reacts with the lysine residues K12, K20, K132, K221, K396, K535, and K537 on BSA^11^. In the corn silk methanolic extract, we isolated and identified several flavone *C*-glycosides with an anti-glycation activity comparable to that of aminoguanidine^10^. As it is known that phenolic compounds such as flavonoids show antioxidation activity, isolated flavone *C*-glycosides could indicate antiglycation activity. Furthermore, we revealed that the corn silk water extract has effects on streptozotocin-induced diabetic nephropathy in rats^12^. However, the active components in the hot water extract remained unidentified. In this study, we aimed to elucidate the structure of the active components that inhibit glycation in the water extract of corn silk, as this herbal extract has great potential as a therapeutic agent to prevent and alleviate diabetic complications and dysfunctions caused by aging. In this research we revealed that water extract of corn silk showed anti-glycation activity and active component was lignin-carbohydrate complex. Lignin is one of the components of the cell walls and is usually a coproduct of paper and pulping, as well as lignocellulosic bioethanol industries^13^. Lignin consists of three basic structural units, *p*-coumaryl alcohol, coniferyl alcohol, and sinapyl alcohol. These structural units are connected by ether and C−C bonds^14^. Furthermore, it is known that lignin shows promising antioxidant activity and is expected as functional material in cosmetics and pharmaceuticals fields^15^. The overview experimental procedure of this research was illustrated in Fig. 1.

## Results and discussion

### In vitro glycation of BSA

First, we examined the inhibitory effect of the corn silk water extract on AGEs formation using a specific enzyme-linked immunosorbent assay (ELISA). The inhibitory activity (%) was 100% at a concentration of 25 μg/mL. In our preliminary research ten corn silk samples available in the market were tested for anti-glycation activities. As the results, the corn silk samples could be distinguished based on their inhibitory activity at the concentration of 25 μg/mL. Thus, the corn silk sample was prepared at 25 µg/mL for ant-glycation assay in this experiment.

### Preparation of > 3 kDa fraction

To explore the active components of corn silk, its aqueous extract was separated into two fractions based on molecular size using a Centricon Plus-70 (3 kDa, Merck Millipore Ltd., Darmstadt, Germany). The inhibitory activities of these fractions were confirmed using an AGEs-specific ELISA. The > 3 kDa and < 3 kDa fractions at 100 μg/mL showed inhibitory activities of 79.1% and 71.1%, respectively. In comparison, the inhibitory activity of aminoguanidine (positive control) was 96.1% at 10 mM (Table 1). As the > 3 kDa fraction showed a slightly stronger inhibitory activity than the < 3 kDa fraction, we only used the > 3 kDa fraction in the following tests. In addition, in order to isolate new active compounds other than the known compounds with small molecular weight^16^ we focused on > 3 kDa fraction.

**Table 1:** Anti-glycation activites of fractions prepared from water extract of corn silk.

	Inhibition rate(%)
> 3 k Da(100 µg/mL)	79.1%
< 3 k Da(100 µg/mL)	71.1%
Aminoguanidine (10 mM)	96.1%

### Reversed phase (RP)-HPLC analysis of the > 3 kDa fraction

**Figure 2:**
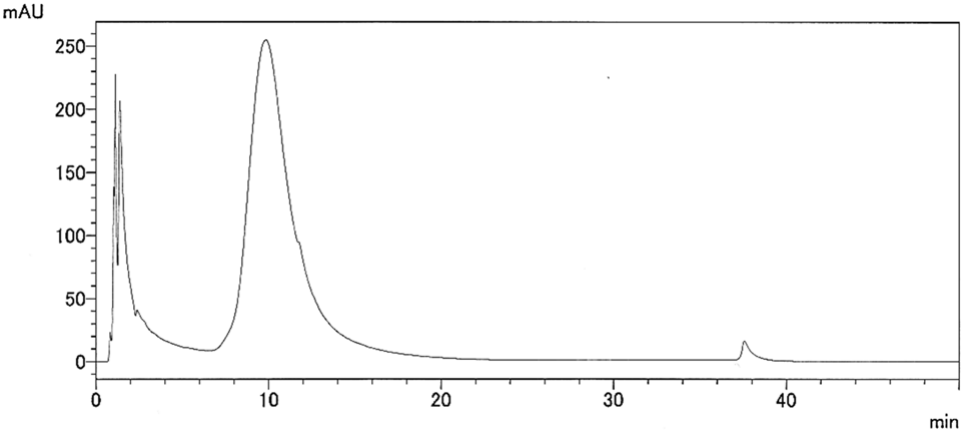
HPLC chromatogram of the > 3 kDa fraction of the corn silk water extract.

In previous studies, polyphenols are known as anti-glycation active compounds in natural herb and foodstuffs^17^. To confirm an existence of high molecular weight polyphenols in the corn silk water extract, the > 3 kDa fraction was analyzed using a RP-HPLC system equipped with a UV detector set at 254 nm. The chromatogram of the > 3 kDa fraction showed a broad peak, similar to a hump (Fig. 2). This pattern has been reported in polyphenols analyses using HPLC^18,19^, which suggested that the > 3 kDa fraction contained high-molecular-weight polyphenols.

### GPC analysis of the > 3 kDa fraction

**Figure 3:**
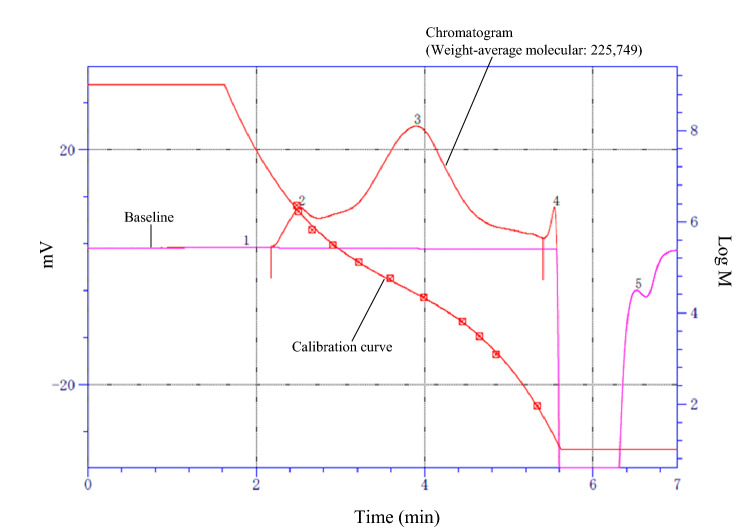
GPC Chromatograms of > 3 k Da fraction of corn silk water wxtract.

In general, the molecular weight of high-molecular-weight polyphenols is difficult to determine because their structures are unspecified, and their sizes are large and wide. Therefore, we attempted to determine molecular weight of the > 3 k Da fraction by gel permeation chromatography (GPC). The GPC analysis showed that the average molecular weight of the > 3 kDa fraction was 225,749 Da (Fig. 3). Subsequently, we performed phloroglucinol and benzyl mercaptan degradations, which are widely used for the structural analysis of condensed tannins^20–24^. Flavan-3-ol units specific to condensed tannins were not detected, and hydrolysable tannins, such as ellagic and gallic acids^24^, were not observed in the acid hydrolysis experiments. According to these results, the active compounds of the corn silk water extract were neither condensed nor hydrolysable tannins.

### Structure elucidation of > 3 kDa fraction

**Figure 4:**
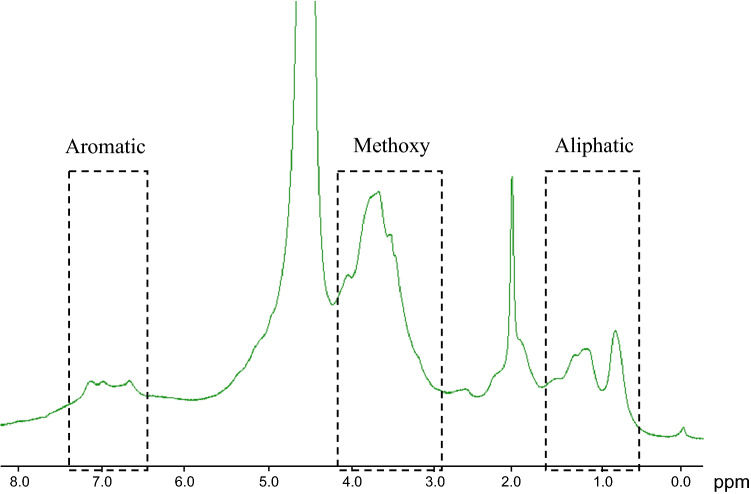
^1^H-NMR spectrum [400 MHz, D_2_O: acetone-*d*_6_ (1:1)] of the > 3 kDa fraction of the corn silk water extract.

**Figure 5:**
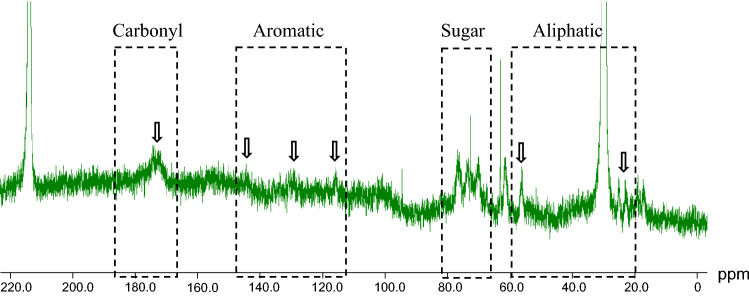
^13^C-NMR spectrum [100 MHz, D_2_O: acetone-*d*_6_ (1:1)] of the > 3 kDa fraction of the corn silk water extract.

**Figure 6:**
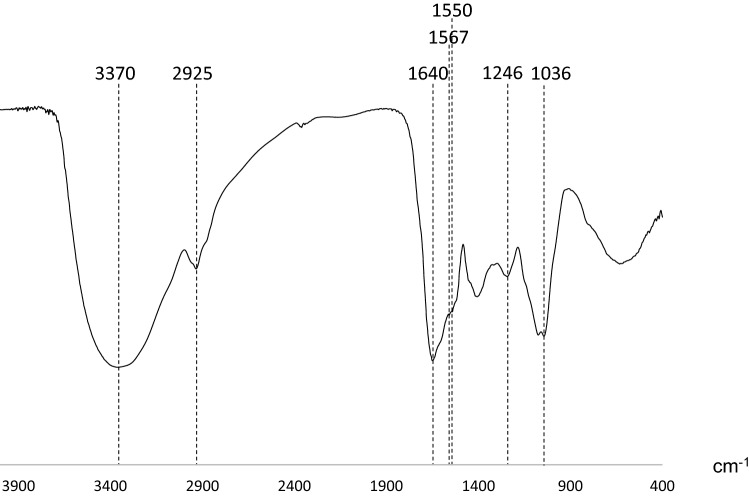
FT-IR spectrum of the > 3 kDa fraction of the corn silk water extract.

**Figure 7:**
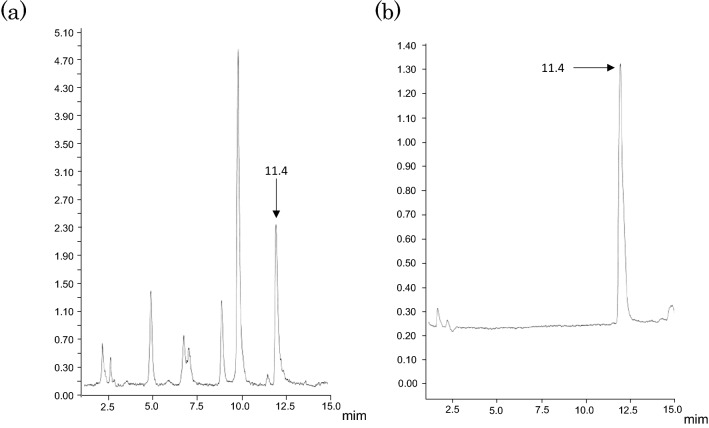
LC–MS chromatogram. (**a**) Aglycons liberated by acid hydrolysis. (**b**) Vanillin.

**Figure 8:**
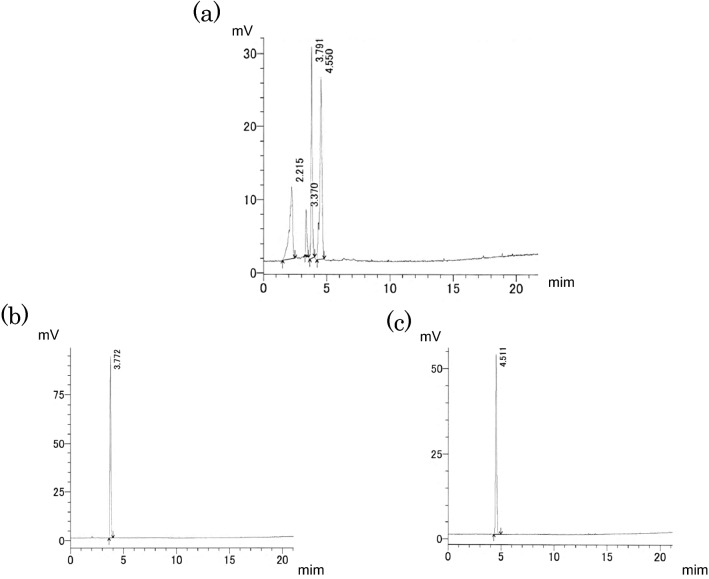
HPLC analysis of sugars. (**a**) > 3 kDa fraction of the corn silk water extract. (**b**) Xylose. (**c**) Glucose.

In order to elucidate the structure of components contained in water extract of corn silk, spectral analyses were conducted such as nuclear magnetic resonance (NMR) and FT-IR spectroscopy. NMR spectroscopy is mainly used to analyze functional groups of organic compounds and to elucidate their structures. The ^1^H-NMR spectrum of the > 3 kDa fraction (Fig. 4) showed signals between 6.5 and 7.3 ppm, which were attributed to aromatic protons. The strong signals observed between 4.0 and 3.0 ppm might be originated from methoxyl protons. Signals attributed to aliphatic moieties were presented between 0.5 and 1.5 ppm. The ^13^C-NMR spectrum of the > 3 kDa fraction (Fig. 5) revealed signals at 23 and 56 ppm, which were attributed to *sp*^3^ aliphatic carbons. In particular, the signal at 56 ppm was considered to be methoxyl carbon. Sugar-derived signals were also observed at 70–80 ppm. In addition, signals at 115, 130, and 145 ppm, attributed to *sp*^2^ aromatic carbons, and signals at 173 and 175 ppm, attributed to carbonyls, were observed in the ^13^C-NMR spectrum. These spectral data suggested that phenolic components were present in large amounts in the > 3 kDa fraction. Moreover, these NMR spectra suggested that the phenolic polymer compounds were lignin. An et al. reported the characteristic NMR spectral data of lignin^15^. Good agreement was obtained between our spectral data and their literature values. Therefore, we aimed to confirm the presence of lignin in the > 3 kDa fraction. In the Fourier transform infrared (FT-IR) spectra, the band at 3370 cm^−1^ was engendered by the hydroxyl group. The band at approximately 2925 cm^−1^ was assigned to the C–H stretching vibration of methyl and methane. The signal at 1640 cm^−1^ was attributed to the conjugated carbonyl group, and the regions at 1567 cm^−1^ and 1550 cm^−1^ represented the characteristic absorption of the benzene skeleton. The band at 1246 cm^−1^ was typical of the guaiacyl unit, and the band at 1036 cm^−1^ was attributed to the stretching vibration of the ether bond in polysaccharides (Fig. 6). The guaiacyl unit is known to be derived from coniferyl alcohol, one of the constituent units of lignin. These results were consistent with the reported lignin spectrum^15^. As the hydrolysis of lignin releases vanillin and sugars^14^, the acid hydrolysate prepared from the > 3 kDa fraction was analyzed by liquid chromatography (LC)-MS to detect vanillin (Fig. 7). In addition, glucose and xylose were identified using HPLC (Fig. 8), confirming the presence of lignin-carbohydrate complex in the > 3 kDa fraction. Although structure elucidation is preferable without degradation being conducted, the degradation process was necessary to obtain partial structure information of high molecular compound such as lignin. It is known that the formation of AGEs is accompanied by the generation of various free radicals and antioxidant protect against glycation-derived free radicals^25^. Lignin is also known to show radical scavenging activity and act as antioxidant^26^. Therefore, one possible explanation for the inhibitory activity of lignin against glycation is its antioxidant capacity.

In summary, we determined that the > 3 kDa fraction of the corn silk water extract contains lignin-carbohydrate complex, which is a phenolic polymer compound including sugar units. Polyphenols such as tannins were previously considered as the inhibitory active compounds against the glycation in corn silk. However, this study revealed for the first time that the active component of the corn silk water extract is lignin-carbohydrate complex. Therefore, our results can promote the application of corn silk as a material for functional foods, by specifying the characteristic constituents of corn silk and suggesting a basis for quality control. We are now planning to develop quality control methods of corn silk available in market using NMR spectroscopy combined with statistical analysis and intend to report it in a later paper.

## Conclusions

In this study we confirmed that > 3 kDa fraction derived from water extract of corn silk showed anti-glycation activity. Furthermore, lignin-carbohydrate complex was identified as active component against glycation. This result can promote the application of corn silk as a material for functional foods.

## Experimental procedures

### Plant material

Corn silk (Lot: 293,011) was purchased from Kojima Kampo Co., Ltd. (Osaka, Japan). The specimens were maintained at the Laboratory of Natural Products and Phytochemistry, Department of Pharmaceutical Sciences, Faculty of Pharmacy and Pharmaceutical Sciences, Josai University.

### Extraction

Dried corn silk (500 g) was extracted using H_2_O for 24 h at room temperature. The extract was filtered through a qualitative filter paper No.2 (Toyo Roshi Kaisha, Ltd., Tokyo, Japan). The remaining water extract was frozen and freeze-dried in FDU2110 (TOKYO RIKAKIKAI CO, LTD, Tokyo, Japan) under − 83 °C at 3.1 Pa pressure for 24 h. A freeze-dried residue (19.7 g) was obtained with a yield of 3.94%.

### Ultrafiltration

The freeze-dried residue (560 mg) was dissolved in H_2_O (280 mL) and centrifuged using a Centricon Plus-70 (3 kDa, Merck Millipore Ltd.) at 2000 × *g* for 60 min. The yields of the resultant fractions with molecular weights > 3 kDa and < 3 kDa were 208.8 mg and 288.8 mg, respectively.

### In vitro glycation of BSA

Bovine serum albumin (4 mg/mL) was incubated with 200 mM glucose in the presence or absence of extract (25 μg/mL) or fraction (100 μg/mL) in 0.1 M phosphate buffer (pH 7.4) for 7 days at 37 °C. After incubation, the level of generated glycated BSA (CML) was measured using a glycated BSA-specific ELISA based on the method described by Suzuki et al.^16^. Briefly, each well of a 96-well microtiter plate was coated with 100 µL of the indicated sample, followed by incubation for 1 h. The wells were washed three times with PBS containing 0.05% Tween 20 (washing buffer), The wells were then blocked with 0.05% gelatin in PBS for 1 h. After triplicate washing with washing buffer, the wells were incubated for 1 h with 100 µL of the specific antibodies for glycated BSA (anti AGEs monoclonal antibody, Clone No. 6D12) (Trans Genic Inc., Fukuoka, Japan). After triplicate washing with washing buffer, the wells were incubated with horseradish peroxidase (HRP)- conjugated anti mouse IgG antibody (Funakoshi Co., Ltd., Tokyo, Japan), followed by reaction with 1,2-phenylenediamine dihydrochloride. The reaction was terminated with 100 µL of 1.0 M sulfuric acid, and the absorbance was read at 492 nm with a micro-ELISA plate reader. Inhibition was calculated as follows: inhibition (%) = [1−(As − Ab)/(Ac − Ab)] × 100, where As is the level of glycated BSA of the incubated mixture with sample, Ab is the level of glycated BSA of the incubated mixture without sample and glucose as blank control, and Ac is the level of glycated BSA of the incubated mixture without sample as a control. Aminoguanidine which was well known synthetic inhibitor was used as positive control at concentration of 10 mM.

### RP-HPLC analysis of the > 3 kDa fraction

RP-HPLC assays were performed using a Shimadzu Prominence HPLC system (Shimadzu Co., Kyoto, Japan) composed of a pump equipped with a degasser, autosampler, column oven, UV detector (detection wavelength set at 254 nm), and evaporative light scattering detector (ELSD)-LT II (Shimadzu Co.). The ELSD parameters were set as follows: drift tube temperature, 40 °C and pressure, 300 kPa. Data were recorded using LabSolutions software (Shimadzu Co.). HPLC assays were conducted using a Senshu Pak ODS column (5 μm, 250 × 4.6 mm i.d., Senshu Scientific Co., Ltd., Tokyo, Japan). The sample concentration was 10 mg/mL. Injection volume, flow rate, and column temperature were 10 μL, 1 mL/min, and 40 °C, respectively. The mixture of solvent A (H_2_O) and B (90% aqueous acetonitrile) was used as mobile phase and eluted with a linear gradient system: 5% solvent B for 3 min, 5–100% B over 15 min, and 100% B for 15 min. Samples were filtered through a 0.45-µm Minisart Syringe Filter (Sartorius Stedim Biotech GmbH, Goettingen, Germany) before HPLC analysis.

### GPC analysis of the > 3 kDa fraction

The GPC system was composed of an SSC-3461 HPLC pump (Senshu Scientific Co., Ltd.), SSC-2320 column oven, and ERC-7517 RI detector (IDEX Health & Science, Oak Harbor, WA, USA). GPC assays were conducted using a TSK-gel Super AW 4000 column (150 × 6.0 mm i.d., Tosoh Co., Tokyo, Japan). The mobile phase was *N*, *N*-dimethylformamide with 0.5% of ammonium formate solution (3 mol/L). The flow rate was 0.6 mL/min. The temperature was maintained at 40 °C. Shodex Standard SM-105 (polystyrene standards, Showa Denko K. K., Minato-ku, Tokyo, Japan) was used for GPC assays. GPC data were analyzed using μ7 Data Station software (System Instruments Co., Ltd., Hachioji, Tokyo, Japan). The sample was dissolved in 50% aqueous *N*, *N*-dimethylformamide at concentrations of 10 mg/mL.

### NMR spectra measurements of the > 3 kDa fraction

One-dimensional NMR spectra were recorded using an Agilent 400MR-vnmrs 400 spectrometer (Agilent Technologies, Inc., Santa Clara, CA, USA; 400 MHz for ^1^H, 100 MHz for ^13^C) at room temperature with solvent signals as internal references. All chemical shifts (δ) were given in ppm, and the sample was dissolved in D_2_O: acetone-*d*_6_ (1:1) at concentration of 100 mg/mL.

### Acid hydrolysis of the > 3 kDa fraction

The > 3 kDa fraction (50 mg) was dissolved in 0.5 N H_2_SO_4_ (10 mL) and heated under reflux for 5 h at 105 °C with magnetic stirring. The mixture was neutralized with Amberlite IRA96SB and lyophilized. The sugars generated by acid hydrolysis were identified by HPLC using standard markers of glucose, xylose, arabinose, sucrose, rhamnose, and fructose, as described in the following section. Aglycones were extracted with ethyl acetate and analyzed by LC–MS. The entire residue was dissolved in 1 mL of MeOH for analysis.

### Sugar analysis

HPLC analysis of sugars was conducted using a Unison UK-Amino column (3 μm, 250 × 3 mm i.d., Imtakt Co., Kyoto, Japan). The sample injection volume was 2 μL, flow rate was 0.6 mL/min, and column temperature was maintained at 60 °C. The mixture of solvent A (H_2_O) and B (acetonitrile) was used as mobile phase and eluted with a linear gradient system: 90% solvent B for 6 min, 90–75% B over 20 min, and 75% B for 25 min.

### LC–MS analysis of aglycons liberated by acid hydrolysis

LC–MS was performed using an SCL-40 system (Nexera series, Shimadzu Co.) composed of a pump equipped with a degasser, autosampler, column oven, SPD-40 UV detector (wavelength set at 254 nm), and LCMS-8050 triple quadrupole mass spectrometer. Data were recorded using LabSolutions software (Shimadzu Co.). HPLC assays were performed using an X Bridge C18 column (5 μm, 150 × 2.1 mm i.d., Waters Co., Milford, MA, USA). The sample injection volume was 1 μL, flow rate was 0.2 mL/min, and column temperature was maintained at 40 °C. The mixture of solvent A (H_2_O) and B (acetonitrile) was used as mobile phase and eluted with a linear gradient system: 5% solvent B for 3 min, 5–20% B over 10 min, and 100% B for 33 min. Mass spectra were recorded in ESI (negative ionization scan mode) between *m/z* 100 and 600 using the following conditions: nebulizing gas flow, 3.0 L/min; drying gas flow, 10 L/min; heating gas flow, 10 L/min; desolvation line temperature, 250 °C; block heater temperature, 400 °C; and interface temperature, 300 °C. The vanillin signal (*m/z* 151, [M–H]^−^) was confirmed on an extracted ion chromatogram.

### FT-IR

Water extraction was characterized by a JASCO FT/IR-410 (JASCO Corporation, Tokyo, Japan) using the KBr tablet method (resolution: 4 cm^−1^, measurement wavenumber range: 400–4000 cm^−1^). Background correction was performed using a single KBr tablet.

## Data Availability

The datasets generated and analyzed during the current study are available from the corresponding author on reasonable request.
